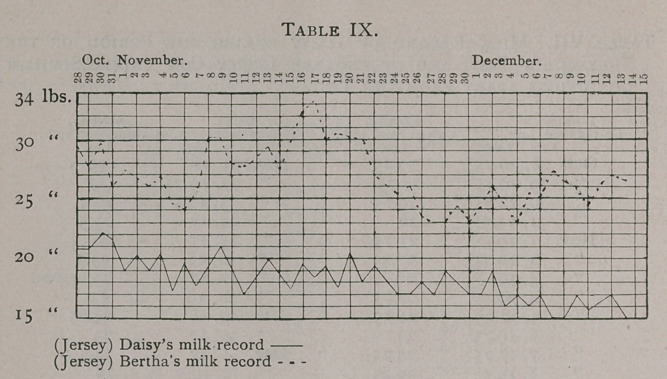# Experiments with Tuberculin on Non-tuberculous Cows

**Published:** 1895-09

**Authors:** James Law

**Affiliations:** Ithaca, N. Y.


					﻿THE JOURNAL
OF
COMPARATIVE MEDICINE AND
VETERINARY ARCHIVES.
Vol. XVI.	SEPTEMBER, 1895.	No. 9.
EXPERIMENTS WITH TUBERCULIN ON NON-TUBER-
CULOUS COWS.
BY JAMES LAW, F.R.C.V.S.,
ITHACA, N. Y.
On October 28, 1894, the following cows were set apart for
this experiment: Two Holstein cows and one Jersey in full flow
of milk, being about six weeks after calving, and two dry far-
row cow.s of common stock, one pointing to a short-horn an-
cestry and the other to a Devon one. Meanwhile observations
on the milk of three other cows, two Holsteins and a Jersey,
about the same length of time after calving, afforded a fair com-
parison between cows treated with tuberculin and others under
similar conditions but without such treatment.
The first five cows to be tested with tuberculin, each received,
in proportion to its size, a full dose of tuberculin weekly, and
the-temperatures were taken before the injection for the normal
standard and about every two hours from about the ninth to
about the twentieth hour after each tuberculin injection.
Temperature. The tested animals were treated like the rest
of the herd with the single exception that, in order to take the
temperatures, they were tied up in the stalls for twenty-four
hours on each occasion for testing, while the others were at
liberty under an enclosed shed except when tied up for feeding
and milking. The prolonged standing on hard boards led on
each occasion to congestion of the feet of the Holstein cow
Mabel, which weighed 1455 lbs., but, as this invariably took
place under similar circumstances and apart from the injection
of tuberculin, the slight rise of temperature on each occasion
of testing is abundantly accounted for from the condition of the
feet alone. This conclusion is further confirmed by the fact
that, excepting in cases in which she was turned out in the
afternoon to relieve her tender feet, the temperature went on
steadily increasing to the last. This was notoriously the case
in the three last tests of the series (Nov. 28th, Dec. 7th, Dec.
12th). On a previous occasion Mabel had been tested in com-
pany with the entire herd and stood the test satisfactorily.
Even in the present series of tests, this cow (with one excep-
tion) never rose more than two degrees above her initial tem-
perature taken when that particular test was started, and she
only rose nine-tenths of a degree above 102° F., which may be
set down as the normal standard temperature of a cow in full
milk, highly fed and kept indoors. Taking into account the
variations in healthy cattle from one time of the day to another,
this rise of less than one degree above the general standard
implies nothing.
The exceptional case was on Nov. 9th and 10th, when Mabel’s
temperature rose to 104° and that of the short-horn grade to
104.30. Taken by itself this test might have been misleading,
but in connection with six other tests (in case of Mabel seven),
made both before and after this, with the same dose of tuber-
culin and with no such resulting rise, it can safely be set down
to accidental conditions. The real cause was not clearly made
out, but it is probable that it was chargeable on exposure in a
cold draught. • Both cows stood on separate ranges close to the
east door of the barn, through which the manure had to be
forked out, and with a cold east wind entering by that door,
and blowing on cattle that had been shut up in a warm build-
ing over night, a slight chill was to be expected.
The Jersey, Daisy, never rose above the normal standard of
102°, excepting in the first test, and then only to 102.30—too
little to furnish even a suggestion of tuberculosis, no higher
than we find in many well-fed healthy cattle.
The Holstein, Belva, on two occasions rose to 102.5 °, half a
degree above the normal standard, but which is often attained
to in health, and apart from the tuberculin test. Moreover, on
five other tests, both before and after these, she did not show a
rise over 102°, so that the less suspicion should arise from this
insignificant elevation.
The Devon grade-cow in different tests had her temperature
elevated to 102° and on one occasion to 102.6°, a little more
than half a degree above the normal, and which, as already said,
is found in the healthiest cows.
The short-horn grade had a fever temperature on one occasion
apparently from a chill, as already referred to. In her first test
it rose to 102.6°, as did also the Devon grade on the same oc-
casion. This may be explained partly by the fact that both had
been driven a distance of seven miles the day previous, causing
much excitement, and followed by the excitement induced by
coming into a new place and herd, and among new people.
One other point should be named as affecting the tempera-
tures of all the test animals in the early forenoon and late after-
noon. The whole herd was put in the barn for feeding and
milking, from five to seven in the morning and from three to
six in the afternoon, so that at these hours the place was
crowded and the disturbance greater. Elevations of tempera-
ture of a degree and under occurring at such time, and as re-
peatedly seen in the tested animals, are thus accounted for.
Such elevations do not show the persistence and the slow gra-
dations of rise and fall which we usually see in the rise caused
by the tuberculin.
Taken all in all, then, there is nothing in the records of tem-
perature that would indicate, either at the time of the test or
later, that the tuberculin had proved in any way inimical to the
general health. Had the health been impaired by the repeated
operation of the tuberculin it might have been expected that the
constitutional disturbance would have been more distinctly
marked in the later tests than in the earlier ones, and as no such
tendency is observable it may be safely concluded that, so far as
illness can be indicated by a variation of temperature, test doses
of tuberculin, in the absence of the bacillus, do not seem to
produce any such illness in the healthy animal.
It has been alleged that the repeated use of tuberculin on
animals slightly tuberculous abolishes the tendency to reaction
under the use of this agent. If this were true, it would argue
rather a curative than a malific action of the tuberculin ; but
in other experiments I have found the second test, made a
week or more after the first, to produce a no less marked reac-
tion, so that this alleged tolerance need not be taken into ac-
count in the cases before us.
Respiration and Pulse. As regards the record of the pulse
and breathing given in the tables it is sufficient to say that they
furnish no real indication of deviation from the most perfect
health. In cattle pulse and breathing vary so widely under
different conditions of the environment, digestive organs, exer-
cise, etc., that it would take very much greater variations than
those given in the tables to give true indications of disease.
Milk Record. The milk record may be accepted as a more
sensitive test of constitutional injury than temperature, breath-
ing, or pulse. It is also further reaching than these other indi-
cations, as it involves a healthy exercise of all the bodily func-
tions, and above all those of appetite, digestion, assimilation,
and secretion. An appreciable disturbance of the health at any
one point will usually be manifested in this delicate balance in a
variation of quantity or quality of the milk.
Belva~ Taking the milk record of Belva as given in Table
VI., we find that the milk-yield in the twenty .hours follow-
ing the injection of the tuberculin shows no constant nor strik-
ing difference from that of intervening days. The highest yield
per day (42.25 lbs.) was on the fifth day succeeding the third
injection of tuberculin, and on each of these five days the yield
was from two to five pounds above the’average. The lowest
yield per day (31.5 lbs.) was on the fourth day after the first in-
jection, while the preceding day’s yield had been over a pound
above the average, and the two days following the injection had
been respectively two and three pounds below.
What is more significant is that the average yield of milk for
the days following the seven injections of tuberculin is practi-
cally the same as the average yield for the whole forty-seven
days included in the experiment. This may be stated clearly
in tabular form thus :
Average of the seven days following the injections of tuber-
culin 37.257 lbs.
Average of the forty-seven days for which the milk record is
given 37.247. The difference is 0.01, and is in favor of the days
when the system was charged with the dose of tuberculin.
Daisy. The milk record of Daisy given in Table VII. shows
a great difference in the yield on different days, but no constant
relation between the low daily yield and the days when the
tuberculin was in the system. On the first, second, and fourth
occasions in which the system was charged with the tuberculin
the milk-yield was above the average, whereas on the third,
fifth, sixth, and seventh occasions it was below. The highest
daily yield (22.25 lbs.) was on the day in the evening of which
the first injection of tuberculin was made, and the second high-
est (21.5 lbs.), only three-fourths of a pound less, was on the day
after that injection. The lowest daily yield (15 lbs.) was on the
day when the last injection of tuberculin was in the system, and
when besides the cow was in heat. This low yield was also
reached on the day preceding the second last tuberculin injec-
tion (the ninth day after an injection), and also on the succeed-
ing day when the system was charged with this second last
injection. This low record could not be justly charged on the
tuberculin injection, seeing that it was already reached the day
before that injection.
Daisy, like the rest of the herd, was falling off in milk during
the experiment, and her average when charged with tuberculin
suffers on account of her having reached her lowest mark on
December 7th, on the evening on which a dose of tuberculin
was given, and, further, that on December 13th, the day* of the
last test, her milk shrank because she was in heat Taking the
seven tests the averages stand thus :
Average of the seven days following the injections of tuber-
culin 17.82 lbs.
Average of the forty-seven days for which the milk record is
given, 18.26 lbs. This shows a difference of less than half a
pound daily on the average against the tuberculin. If we leave
out the last injection (Dec. 13th), when the cow was in heat, we
find that the average yield per diem for the six days during which
the cow was charged with tuberculin is slightly above the aver-
age for the whole forty-seven days of the trial.
Molly, Freda, and Bertha. These cows were not injected with
tuberculin, and their milk records have been introduced to show
that the daily oscillations in the yield and its progressive diminu-
tion in the main during the forty-seven days was common to the
whole herd, and in no sense peculiar to the three cows that had
been treated with tuberculin. The gradual failure can be seen
in the tables. It may be more clearly shown by placing side by
side the general average for the first four weeks and the average
for the last two weeks and five days.
Average for first twenty-eight days :
Belva
lbs.
38.10
Molly
lbs.
41.31
Freda
lbs.
43.51
Daisy
lbs.
19 23
Bertha
lbs.
28.46
Average for last nineteen days :
Belva
lbs.
36.OO
—2.IO
Molly
lbs.
42.87
+ 1.56
Freda
lbs.
40.51
—3-oo
Daisy
lbs.
16.76
—2-47
Bertha
lbs.
26.13
—2-33
Molly has gone on improving, but the others show a very de-
cided falling off, which is greater in the nort-injected Freda than in
the injected Daisy, and greater in the uninjected Bertha than in
the injected Belva.
Oscillations. The variations above and below the general
average for each animal injected and not injected with tubercu-
lin will be very clearly seen by glancing at the tables giving
the graphic illustration for two Holsteins and two Jerseys
(Tables VIII. and IX.). In figures they may be shown as follows:
Belva. General average per day 37.247 lbs. Highest per
day 42.25 lbs. Lowest per day 31.50 lbs.
Freda. General average per day 41.78 lbs. Highest per day
47.50 lbs. Lowest per day 34.50 lbs.
Daisy. General average per day 18.26 lbs. Highest per day
22.25 ^s. Lowest per day 15 lbs.
Bertha. General average per day 27.01 lbs. Highest per
day 33.5 lbs. Lowest per day 23 lbs.
Molly. General average per day 41.19 lbs. Highest per day
48 lbs. Lowest per day 33.5 lbs.
The extremes, it will be observed, were actually greater for the
cows that were not treated with tuberculin than for those so
treated Among the Holsteins, Belva had a variation amounting
to 10.75 lbs., Freda one of 13 lbs., and Molly one of 14.5 lbs.
Among the Jerseys, Daisy had a variation of 7.25 lbs. and Bertha
one of 10.5 lbs. Extreme variations in the yield of milk then
cannot be charged on the action of a test dose of tuberculin in-
jected into a healthy animal, nor of a series of such test doses
administered at intervals of a week.
Percentage of Butter-fats in the Milk. Before dismiss-
ing the milk it is desirable to consider how the ratio of butter-
fats is affected by repeated test doses of turberculin injected into
a healthy animal. A study of the tables given below will fail to
establish any connection between the presence of a test dose of
the tuberculin in the animal body and any increase or diminu-
tion of the fat in the milk. The Holstein Belva had her highest
percentage of butter-fat (3.6) October 20th, ten days before the
first injection of tuberculin. Her next highest record (3.4) was
Dec. 8th, while under the action of tuberculin. Her lowest rec-
ord (2.8) was Dec. 1st, two days after the operation of a dose of
tuberculin. Her variation (0.8) is only a little more than that
of the untreated cow Freda (0.6), and only about half that of
Molly (1.5). The Jersey Daisy also made her highest percent-
age (5.6) October 20th and her lowest (4.8) December 8th, when
under the action of tuberculin. But she made her second highest
(5-55) Nov. iotfi, when under tuberculin, and an equal record
Nov. 17th, two days after the operation of a dose of tuberculin.
Her greatest variation was 0.7 per cent., whereas that of the un-
treated cow Bertha was 1.15 per cent.
There is therefore no change in the percentage of butter-fats
sufficient to indicate any disease or ill-health as the result of the
administration of repeated test doses of tuberculin.
Effect on Body-weight. The weight of the animals varied
so little during the experiment that it might be said to have re-
mained stationary. The record is as follows :
October 30th.
Belva, 1264 lbs.,
Mabel, 1455 “
Daisy, 945 “
Short-horn Grade,
Devon Grade,
Dec. 1st.
1305 lbs.,
1540 “
950 “
IO2O “
895 “
Dec. 13th.
1020 lbs.,
915 “
Jan. 5, 1895.
1405 lbs.
1570* “
965 “
1025 “
910 “
Considering that a variation of 50 lbs. in the weight of a cow
may occur in a few hours according as it is taken before or after
feeding and watering or milking, there may be said to have been
no change excepting in the case of the two Holsteins, in which
there is shown a gain of 141 lbs. and 115 lbs., respectfully. It is
worthy of notice that the last weighing, which makes the highest
record, was made three to four hours after the morning feeding,
and (in the case of the three first cows) of the morning milking.
The two dry cows had been watered but had not been fed on the
morning of the last weighing, as they were just about to be
killed.
It may be concluded that the repeated test doses of tuberculin
had in no injurious way affected assimilation, and that in the two
Holstein cows it had not prevented a perceptible improvement
in this repect.
Post-mortem Examinations. To complete the record the
two farrow cows were killed Dec. 5, 1894, and subjected to
careful necropsy. In the main the viscera were sound. The
short-horn grade had pus in each of the left quarters of the
mammary gland in the milk sinus, the walls of which were red
and thickened. When stained and placed under the microscope,
the pus showed numerous cocci but no bacilli.
As is usual in old cows, the groups of lymphatic glands in the
intermaxillary and pharyngeal regions, in the chest, the abdo-
men, the subcutaneous and intermuscular regions were pig-
mented, of a dark grayish color, varying at different points, but in
no case showing molecular degeneration, coagulation-necrosis
(caseation), nor even perceptible congestion. In the short-horn
grade the lymphatic glands behind the diseased mammae were
considerably enlarged.
Experiments at the U. S. Bureau of Animal Industry.
In the “ investigations concerning bovine tuberculosis, 1894,”
Dr. de Schweinitz records the effect on the milk of two healthy
cows, one of which received one dose and the other three succes-
sive doses of tuberculin. The dose on each occasion was 2 c.c. for
each cow, and, as they were common stock, it may be inferred that
it was a full dose considering the probable weight of the animals.
Of variations in temperature it is enough to say that there
was no more than would occur in the best of health. The anal-
ysis of the milk is given in Table XII., from which it will be
seen that the single test of cow No. 113 there was a slight re-
duction of the total solids and of the different constituents, such
as sugar, albuminoids, and fat. The second cow. No. 217, tested
three times under tuberculin and once on five successive days
without tuberculin, gives a more trustworthy basis for estimating
the effect of that agent. It will be observed that on April 1st
under the tuberculin there was a slight decrease of the total
solids (0.45), on April 13th under tuberculin a still larger de-
crease (1.26), but on June 5th under tuberculin there was an in-
crease (1.01). On June nth to 15th without tuberculin there
was a variation in the total solids of 1.99.
Then, as to the milk sugar, 217 showed a percentage reduction
of 0.1 April 1st under tuberculin and of 0.61 April 13th, but no
change whatever June 5th, though again under tuberculin, and
no change June nth to 15th without tuberculin.
Of albuminoids, 217 showed a percentage reduction of .07
April 1st under tuberculin, but an increase of 0.13 April 13th
and 0.61 June 5th. In the absence of tuberculin it showed a
variation of 0.42 June nth to 15th.
In fat, No. 113 had a decrease in her single test, while 217
had an increase in all cases under tuberculin 0 31 April 1st, 0.13
April 13th, and 0.86 June 5th. In the entire absence of tuber-
culin, June nth to 15th, she showed a variation of 0.51.
With such a testimony it would be disingenuous to claim any
constant or appreciable variation as the result of the injection of
a test dose of tuberculin into a healthy animal, even if such dose
were repeated several times. So far as there is evidence before
us, everything points to the harmlessness of a single test dose on
a sound animal system.	*
Table I. Action of Tuberculin on Holstein Cow Belva, in Full Milk (calved September 16, 1894).
Before injection.	After injection.
•* "V* g b I x I o x * l<	x I o	cl X 4)	•“*	O. X 1 O L. CL X <L I ts cl X 4) L*	cl lx •—
g o\	Eg	S' J2	S	3	g	S'	«	3	8	S'	J2	3	g	S' J2	3	8	S' <n	3	8	S' w	3	8	S'	8
fl 00	O U	v M	O	O	4)	4,	fl	O	4>	4,	fl	O	4,	4)3	O	4,	4, “g	O	4>	4> fl	O	4,	<8	3
Q H	Z **	cd ci,	Q	X	h	nd	cl,	X	b	cd	Ql	X	b	cd cl,	X	b	Pi cl.	|	X	b	cd b	X	b	|b j	X
Oct.	m. P. M.	A. M.	AM.!	A. M	P. M.	P. M.	P. M.
30-31	ioi°	..... 30	9.45	102.1°	...	,..	6.45	ioi.5°3O72	9.15	101.5° 30	60	11.25	102.3°	15 ...	1.45	ioi.9°2o69	4.15	102°	...	6.45
Nov.
9-10	101.3	..... 30	10.00	IOI.8	16	...	7.00	*100.2	13...	930	101.5	... 11.30	1017	17...	2.00	101.4	18...	4.00	IOI	20	6.30
14-15	101.0	15	...	30	10.30	101.6	18	..	7.30	100.8	16 ...	9.30	101.2	16...	11.30	101.5	15 ...	1.00	101.8	13 ...	3.00	102.5	20	5.00
A. M.	p. M.	P. M.	P. M.
23	IOI.O	24	...	30	7.00	101.5	16	...	2.30	IOI.6	21 ...	4.00	101.5	17	...	7.00	IOI.O	I	15 ...1	10.00
P. M.	A. M.	a. M.	A. M.
28-29	100.0 13... 30 10.15	101.8 18... 7.00 100.5 14...	9.15 IOI.O 16... II.45	101.9 2060 2.00	102.5 14... 4.00
Dec.	P. M.
7-8	101.5 20... 30	10.30	101.9 2172	730	1004 1272 10.00	100.9 2060 12.10	IOI.8	1666	3.00	1016	20...	4.45
Was in heat
12-13'	100.8 2063 30	10.30	101.5 16...	7.15	101.4 16... 10.15	101.3 1660 12.15	101.6	18...	2.15	101.7	......	4.30	Dec. nth.
Table II. Action of Tuberculin on Holstein Cow Mabel, in Full Milk (calved October, 1894).
• Before injection.	After injection.
-	g P-	• 1	•	>-<	Cu	X V	o*	« fl)	d.	X	O	O*	X	ft,	H	P<	X	O	H	O.	x	4*
£ O'	Eg	8- J3	«	2	g	g- m 5	g	g- « g	g	g-	«	g	g	§■	«	g	g	g-	*	g	g	g-	g
c« °°	O. 4)	<u 5	o	.9	5	05.2	5	85O	u	<3	S	O	O	<U	P	°	<U	<U	3	.	O	<U	41	9
Q w z *-	Q K E-I 0$ CU X B 0! »< K b <X .cl X h^clX b b X E- a: X
Oct	m. P. M.	A. M.	A. M.	A. M.	P. M.	P. M.	P. M.
30-31	IOO.90	..... 31	9.45	IOO.90... 9.45	102.6° 28	...	9.15	102.9°	42 72	II.25	102.6°	32	...	I.45	102.2° 26	72	4.15	101.5°...	6.45
Nov.
9-10	101.4	.. 31	10.00	102.8	18	...	7.00	104.0 22	..	9.30	102 8	26 ...	II.45	102.6	l8	...	2.00	IOI.7 22	...	4 OO	IOI.5 I9	6.30
14-15	IOI.O	23 ...	31	10.30	101.5	■?	...	6.46	101.9 16	...	8.30	IOI.8	18 ...	II.15	102.0	18	...	1.00	102.2 21	...	3.OO	102.0 20	5-00
A. M.	P. M.	I	P. M.	P. M.
23 IOI.9 22... 31	7.00	102	l8...	230	102-5 |2I ... 4.OO 102.2 24... 7.OO 102.0 22... 10.00
P. M.	A. M.	II	A. M.	A. M.
28-29	101.5 14...	31	10.15	102	21 ...	7.00	100.9 16... 9.15	101.9	14... 11-45	101.7 14...	2.00	102.2	l8 ...	4.OO	102.5	24 5.20
Dec.	I	p. M.	JI
7-8	IOI.4 l8 ...	31	IO.3O	102	20 ...	7.3O	IOI.6 24 ... 10.00	102.3	20 ••• 12-10	102.8 l6 ...	3.OO	102.6	12 ...	4.45
Feet sore from
12-13	102.0 24 63 31	10.30	102.6 18 ... 7.15 102.6 24... 10.15	102.9 16 60 12.15	103.2 31 ...	2.15	103.0 .. 4.30 standing on
boards.
Table III. Action of Tuberculin on Jersey Cow Daisy (calved September 12, 1894).
Before injection.	After injection.
§ g" 9-	I S p" £•”' s S’ bIjS I S &	9-«I S	S’ 3	9-J/j p	gj- s3
QM 2* «£ Q X b cd cu x b cd cl | X h cd [cl | X b cd a ffi b cd [cl M b cd | EC
Oct.	m. P. M.	A. M.	A. M.	A. M.	P. M.	P. M.	P. M.
30-31 ioi.o°........ 26	9.45	101.8°...... 645	101.5° 30 72 9-I5 101.5° 30 60 u-25 102.3° 15 ... 1.45	101.9° 20 69 4-x5 IO2° 6.45
Nov.
9-10	101.0	......	26	10.00	101.6	2472	7.00	100.8	12...	9.30	101.2	17...	11.45	101.7	20...	2.00	101.8	22...	4.00	101.2	18	6.30
14-15	100.8	21 ...	26	10.30	101.8	23 ...	6.45	100.9	9	•••	9-3°	101.0	16 ...	11 15	101.0	17 ...	1.00	101.2	22	...	3.00	101.7	20	5.00
22-23	101.0	18 72	26	10.20	101.5	21 ...	7.00	101.0	15	66	9.30	100.8	15 ...	11.30	101.3	15 ...	2.30	101.5	1	...	4.00	101.3	24	7.00
101.4 ... 10.10
28-29	ioi.o	15 ...	26	10.15	101.7	15 ...	7.00	100.5	16	•••	9-15	100.5	18 ...	ii-45	101.2	14 48	2.00	101.0	14	...	3.00	101.5	...	5.20
Dec.	P. M.
7-8	101.5 27 ...	26	10.30	101.8	30 54	7.30	100.9 18 62 10.00	101.3 22 68 12.10	ioi.o	20 60	3.00	101.8 16 ...	4.45
In heat: took
12-13	101.3 29 66	26	10.30	101.7	16...	7.15	100.3 18 60 10.15	100.7 30 60 12.15	101.8	30...	2 15	101.8 ......	4.30	bull.
____________________I -I _____________________________________ ]	______________________________________________________________'
Table IV. Action of Tuberculin on Grade Short-horn Cow; Nearly Dry; Farrow.
Before injection.	After injection.
’ i ’	?	~ ’	'	'	’	'	' ~i ~
~ <*	£ E	g- ?	g	5	g	gw	0	g	g- w	s	g	g- w	5	g	g-w	£	g	gw	v g	g w Is
aj °2	O 0)	u ;3	o	O	0;	0) £	°	<D	u'S	°	(D	V 3	,9	<L>	<D	.9	0)	(D	O	4> 3	®
Q	04 Q SC E-* Oh SC ^q^PhSC	Ph SC £-< cu SC	Ph cu SC	Pm SC
Nov.	m. A. M.	P. M.	P. M.	P. M.	P. M.	P. M. o	P. M.
3	100.8°....... 28	6.30 101.2° 20-...	2.00 102.3° 17 •■•	400	IO2.6°I. 5.15	102° 21 48 7.OO 101.3° 13 45 9.OO IOI.5 42 48 IO.15
P. M.	A. M.	A. M.
9-10	102.7	— I—	29	IOOO	104.3	3° 72	7.00	1037	22...	9.15	103.8	17...	11.20	102.8	18...	2.00	IO3.O	22...	4.OO	IOI	l6 ...	6.45
A. M.
14-15	101.0	16 54	28	10.30	101.0	2048	7.00	101.7	2056	9.30	101.0	1850	. 101.5	1342	i.00	101.5	1654	3.00	102	1654	5.OO
A. M.
22-23	100.6	21 ...	28	10.20	100.8	25...	7.00	102.0	2554	9.30	102.0	15 60	n.30	101.6	i860	2.30	102.4	2048	4.00
28-29	101.o	16 43	28	10.15	101.3	2044	7.00	102.0	1648	9.15	101.9	1548	1145	101.5	1852	2.00	101.5	1646	4.00	102	. 5.20
Dec.	•	P. M.
7-8	101.5 22 48 28	10.30	100.5 23 5°	7-3°	101.0 22 42 10.00	101.3 16 58 12.10	102.0 16 52	3.00	101.6	18 ... 4.45
12-13- 101.6 3060 29	10.30	101.9 18...	7.15	102.3 1752 10.15	101.7 1852 12.15	101.8 23...	2.15	101.8	.... 4.30
11"	__________________________________________________________r T_______________________________________________
Table V. Action of Tuberculin on Grade Devon Cow; Dry; Farrow.
Before injection.	After injection.
3 00 O 0)	8 13 O O '	®	° L§ S »2	°	$3 O 0) u 3	° P	M	3	5
AM|!z;~ cd (£q| M b cd Cd|E b |cd |cl | M h cd cu K H [cd |cu | SC b cd cl SC b cd a. SC
Nov.	m. A. M.	P. M.	P. M.	P M.	P. M.	P. M. o	P.M.
3	101.5°........ 28	6.30 IOI° 18...	2.00	102.30 17 ...	4.00	102.6° 18 ... 5.15° 102.6° 14 70 7.00 101.9° 14 64 9-°° 101.81564 10.15
P. M.	A. M.	a. M.	A. M.
9-10	101.7	... .	27	10.00	101.8	24 60	7.00	IO2.I	12 ...	9.15	102.2	II ...	11.20	IOI.4	l6 ...	2.00	IOI.4	14 ...	4.OO	IOI.J	14 ...	6.45
14-15	101.5	1862	26	10.30	102.2	2248	700	102.2	1850	9.30	102.2	12 46!	......	IOI.3	I4 48	1.00	IOI.7	l6 4O	3.OO	IOI.8	24 48	5-00
I A. M.
22-23	100.2	14...	27	10.20	IOI.O	15...	7-00	102.0	II 48	9.3O	IO2.0	I844	II.3O	IOI 8	l8 48	2.3O	IOI.5	12 40	4.OO
28-29	IOI.2	1150	27	10.15	102 0	1642	7.OO	101.2	II42	9.15	102.2	IO 42	II 45	IOI.2	14 48	2.00	IOI.O	l8 46	4.OO	IOI.O	......	5.2O
Dec.	P. M.
7-8	1015 1660	27	10.30	101.8 2360	7.30	102.3	24 60 10.00	102.0	1658 12.10	1018 1654	3.00	IOI.6 14...	4.45
12-13	IOI.O 1348	27	10.30	101.7 14 43	7.15	IOI.i	174810.15	10T.3	14 44] 12.15	101.5 16 ...	2.15	101.6 ......	4.30
Table VI. Milk Record of Belva for the Period of the
Tuberculin Test, and of Two Other Holstein Cows in Similar
Conditions apart from the Test.
BELVA.	MOLLY.	FREDA.
Calved Sept. 16th.	Calved Oct 4th. Calved Aug. 28th.
Lbs.	Lbs.	Lbs.
Oct.	28,	40.5	39.5	44.5
“	29,	39.75	42.25	44.5
“	3°>	40.5	42.25	44.5
“	31.	351	37-5	45-25
Nov. i, 34.5	40.75	36.
“	2,	38.5	42.5	41.75
“	3.	3i*5	34-75	40.25
“	4,	37.	34-75	42.75
“	5.	32	33 5	40.5
6, 36	35.75	41.5
“	7,	35-75	37	45-75
“	8,	38.25	40.5	47.5
“	9,	38	40.5	46
“	10,	38	41.25	43 5
"	11,	39	41.5	42.5-
“	12,	36.75	4075	41.75
“	13.	41-25	45-5	45
“	14,	41.5	42.25	47
“	15,	41.251	46.5	4575
“	16.	39-5	43-5	42
“	17, 40.75	'	45	43-75
“	18,	41	45	43-25
“	19,	42.25	48	43.25
“	20,	38	42.5	42
“	21,	37.75	43	4i-5
“	22,	37.25	44.5	37 served.
“	23,	38 51	43-5	37-75
“	24,	37.75	40	37-5
“	25,	36.25	38	34.5
“	26,	34.25	38.5	35.25
“	27,	36.25	41	36
“	28,	35	41	36.25
“	29,	351	41	39
“	3°.	34 75	41-5 served	41.25
Dec. 1,	35.5	42.5	42.25
“	2,	33.75	42	41.5
“	3.	35-75	4i 5	42.25
“	4,	34-75	38.75	40
“	5.	38-25	42.25	43-75
“	6,	39	42.75	44-75
“	7,	38-5	43 75	45
“	8,	34.751	43-25	42 75
“	9,	37 25	43-25	42
“	10.	37.25	41.25	41.5
“	11,	35-75	4i-5	4o
“	12,	3425	42.25	40.5
“	13,	37.251	43	39-5
1 Indicates the 20 hours following the different tuberculin injections.
Table VII. Milk Record of Daisy during the Period of the
Tuberculin Test, and of another Jersey Cow under Similar
Conditions apart from the Test.
DAISY.	BERTHA.	DAISY.	BERTHA.
Calved Sept. 12th.	Calved Sept. 17th. Calved Sept. 12th. Calved Sept. 17th.
Lbs.	Lbs.	Lbs.	Lbs.
Oct. 28, 20.75	29.25	Nov. 21, 18	30
“	29,20.75	27.75	“ 22, 19.25	27
“	30,	22.25	29.75	“	23,	18.51	26.5
“	31,	21.51	26	“	24,	T7	25.5
Nov.	i,	19	27.75	“	25,	17	26
“	2,	20.25	27.25	“	26,	l8	23.5
“	3,	19	26	“	27,	17	23 served
“	4,20.5	26.75	“	28, 19	23
“	5, 17.25	24.75	"	29, 181	24.25
“	6,	19.75	24	“	30,	17	23
“	7,	I7-75	26.25	Dec.	i,	17	24.5
“	8,	19.5	30-25	“	2,	19	26
“	9,	21	30.25	“	3,	16	25.5
“	10,	19.251	28	“	4,	17	24
“	n,	17	27.5	“	5,	16	26.5
“	12,	18.5	28.5	“	6,	17	26
“	13,20	29.75	“	7,15	28.25
14, 19	.	27.75	“	8, 151	27.5
“	15,	17.51	29	"	9,	17	27
“	16,	19.75	32.5	“	10,	15.75	24.25
“	17,	18.25	33.5	“	11,	16.75	26.25
"	18, 19.5	30	“	12,17	27
‘	19,17-5	3°-75	“	13,	151 served 26.75
“	20.20.5	25.5
1 Tuberculin in system, the day following injection.
Table X. Percentage of Butter-fats in Milk of Belva during
Experiment; also in Holstein Cows Molly and Freda not
Injected.
BELVA. MOLLY. FREDA.
Per cent. Per cent. Per cent.
Oct. 20,	3.6	49	3.2
“ 27,	2.85	3.8'5	3.5	Three	days before first injection of
tuberculin.
Nov. 3,	3.2	•	4.25	3.55	Three	days after tuberculin injection.
“	10,	3.2	3.8	3.35	Under tuberculin. Injected night
before.
“	J7»	3«3	3-75	3	Two days after tuberculin.
“	24,	2.9	3.65	3.3	One day after tuberculin.
Dec.	1,	2.8	3.95	2.95	Two days after tuberculin.
8,	3.4	3.4	3.1 | Under tuberculin. Injected night
before.
Table XI. Percentage of Butter-fats in Milk of Daisy during
Tuberculin Experiment; also of Jersey Cow Bertha not
Injected.
DAISY. BERTHA.
Per cent. Per cent.
Oct. 13,	5.1	4.8
“ 2O«	5-6	4-45	'. . .	, v
“ 27,	5.1	5.05	Three days before first injection of tuberculin.
Nov. 3,	5.3	5.05	Three days after tuberculin injection.
“	10,	5.5	5.3	Under tuberculin. Injected night	before.
*'	17,	5.5	5.4	Two days after tuberculin.
“	24,	5.4	4.25	One day after tuberculin.
Dec. 1,	5.05	5.1	Two days after tuberculin.
“	8,	4.9	4.85	Under tuberculin. Injected night	before.
Table XII. Percentage Variation in the Constituents of the
Milk of Healthy Cows Under a Test-dose of Tuberculin.
Acidity..
No. of	Total	Album!-	Ash in Lactic
Animal. Date.	solids.	Sugxr.	noids.	Fat.	milk. acid.
113,	March	31,	11.01	417	3.26	2.54	0.775	Before	injection.
113,	April	i,	10.69	3-84	3-20	1.52	0.696	After
217,	March	31,	10.83	4	2.96	2.23	0.723	Before
217,	April	1,	10.38	4.16	2.89	2.54	0.700	After
217,	“	12,	11.03	4-*7	1.26	2.56	0.681	Before	“
217.	“	13.	9-77	3-57	1-39	i-53	°-727	After
217,	May	31,	12.03	4.16	2.82	2.43	0.711	Before
217,	June	1,	10.25	4.16	2.29	1.27	0.666	“	“
217,	“	5,	11.26	4.16	2.70	2.03	0.688 0.176 After
217,	“	11,	11.97	4.16	4.17	2.03	0.590	No
217,	“	12,	10.82	4.16	3.83	1.52	0.692	“
217,	“	13,	11.30	4.16	3.97	2.02	0.751	“	“
217,	“	15,	11.62	4.16	4.25	203	0.767	“
%
				

## Figures and Tables

**Table VIII. f1:**
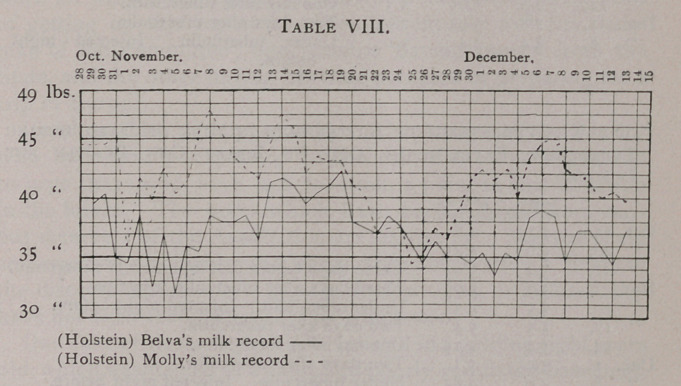


**Table IX. f2:**